# Inhibition of the transient receptor potential melastatin-2 channel causes increased DNA damage and decreased proliferation in breast adenocarcinoma cells

**DOI:** 10.3892/ijo.2015.2919

**Published:** 2015-03-06

**Authors:** MANDI M. HOPKINS, XIAOXING FENG, MENGWEI LIU, LAUREN P. PARKER, DAVID W. KOH

**Affiliations:** 1 Department of Pharmaceutical Sciences, Washington State University, Pullman, WA 99164, USA; 2 Department of Pharmaceutical and Biomedical Sciences, Ohio Northern University, Ada, OH 45810, USA

**Keywords:** transient receptor potential melastatin-2, breast cancer, DNA damage, ion channels

## Abstract

Transient receptor potential, melastatin-2 (TRPM2) is a plasma membrane cation channel with important roles in sensory functions and promoting cell death. However, we demonstrated here that TRPM2 was present in the nuclei of MCF-7 and MDA-MB-231 human breast adenocarcinoma cells, and its pharmacologic inhibition or RNAi silencing caused decreased cell proliferation. Neither an effect on proliferation nor a localization of TRPM2 in the nucleus was observed in noncancerous HMEC and MCF-10A human mammary epithelial cells. Investigation of possible effects of TRPM2 function in the nucleus demonstrated that pharmacologic inhibition or RNAi silencing of TRPM2 in MCF-7 and MDA-MB-231 human breast adenocarcinoma cells caused up to 4-fold increases in DNA damage levels, as compared to noncancerous breast cells after equivalent treatments. These results indicate that TRPM2 has a novel nuclear function in human breast adenocarcinoma cells that facilitates the integrity of genomic DNA, a finding that is distinct from its previously reported role as a plasma membrane cation channel in noncancerous cells. In summary, we report here a novel effect promoted by TRPM2, where it functions to minimize DNA damage and thus may have a role in the protection of genomic DNA in breast cancer cells. Our study therefore provides compelling evidence that TRPM2 has a unique role in breast adenocarcinoma cells. Accordingly, these studies suggest that TRPM2 is a potential therapeutic target, where its pharmacologic inhibition may provide an innovative strategy to selectively increase DNA damage levels in breast cancer cells.

## Introduction

Breast cancer remains the second leading cause of cancer deaths among women. The troubling mortality rates of breast cancer patients, along with the continued incidence of new breast cancer diagnoses each year, illustrate a critical need for new therapeutic targets and improved therapies in breast cancer treatment. Emerging therapeutic targets potentially reside in the transient receptor potential (TRP) superfamily of cation channels. Recent studies have demonstrated important roles for TRP channels in several types of human cancer (1–3). However, little is known regarding the role of these cation channels in breast cancer. Determining the role of TRPs in breast cancer may help identify novel molecular targets for the treatment of breast cancer patients and thus help reduce the mortality rates of this devastating disease.

The TRP superfamily is a diverse set of cation channels that facilitate a variety of cellular functions. The largest TRP subfamily is the TRP melastatin (TRPM) set of cation channels. TRPM channels are known to mediate sensory and adaptive functions, such as taste, thermosensitivity, and touch (4,5). TRPM2 is a unique member of the TRPM subfamily, a widely expressed, non-selective cation channel that also possesses adenosine diphosphoribose (ADP-ribose) pyrophosphatase activity (6). The binding of ADP-ribose leads to the enzymatic activity and the opening of this ion channel. Thus, upon activation of this ‘chanzyme’ by ADP-ribose, cations are gated into the cell. Most notable of these cations is calcium, where the influx of calcium in response to oxidative stress leads to the calcium-mediated activation of pro-cell death apoptotic (7) and non-apoptotic proteins (8,9). TRPM2 thus appears to facilitate the progression of caspase-dependent and caspase-independent cell death mechanisms after oxidative stress (10). Accordingly, activation of TRPM2 has been shown to exacerbate the injury that occurs in response to oxidative stress in noncancerous cells, including neuronal (11), pancreatic (12), and hematopoietic cells (9). Pharmacologic inhibition of TRPM2 was subsequently shown to decrease cell death in these instances, as well as increase cell survival in several other cell lines and tissues (13–15). The rationale for pharmacologically inhibiting the activation of TRPM2 is based upon the ability of TRPM2 inhibitors to decrease the cell death and tissue injury that occurs due to debilitating diseases and conditions. Taken together, the current knowledge of TRPM2 has provided the basis for the development of pharmacologic inhibitors of TRPM2 in order to treat debilitating conditions that involve excessive cell death, including stroke, diabetes, immune disorders and inflammation.

Since TRPM2 has mostly been investigated in noncancerous cells, less is known about the function of the TRPM2 cation channel in cancer cells. Two TRPM2 mRNA transcripts, one antisense transcript and one truncated TRPM2 transcript, were shown to be increased in 80% of metastatic melanoma cell lines (16). Functional analysis of the protein products of these transcripts demonstrated that overexpression of wild-type TRPM2 or knockout of the truncated TRPM2 transcript increased cytotoxicity in melanoma cells. Similarly, RNAi silencing of TRPM2 in prostate cancer cells decrease their proliferation, which suggests that TRPM2 has a role in facilitating prostate cancer cell propagation and growth (17). In this same study, it was shown that TRPM2, normally localized to the plasma membrane or lysosomal membrane (7,12), was localized to the nucleus in prostate cancer cells.

To date, no additional evidence has been reported that shows a nuclear localization of TRPM2 in any other cancerous or noncancerous cell line. Further, other TRPM subfamily members have been shown to have novel roles in cancer. For example, TRPM8 was shown to be upregulated in pancreatic adenocarcinoma cells and vital to their proliferation (2). In summary, TRPM channels appear to have novel roles in various types of human cancer. It is thus possible that the unique member, TRPM2, may also have a novel role in human cancers as well.

In this study, our objective was to investigate TRPM2 in two lines of human breast adenocarcinoma cells in order to analyze its function in breast cancer cells and to provide a preliminary evaluation of its potential as a pharmacologic target in breast cancer. We show here that inhibition of TRPM2 function causes decreased proliferation and increased levels of DNA damage in breast adenocarcinoma cells, with minimal effects in normal breast cells. Because these results have not been previously reported in breast cancer cells, our studies present preliminary evidence that TRPM2 is a potential therapeutic target in breast cancer and its pharmacologic inhibition is expected to selectively target breast cancer cells.

## Materials and methods

### Cell lines and cell culture reagents

HEK 293 (human embryonic kidney), MCF-10A (human mammary epithelial), MCF-7 (human breast adenocarcinoma), and MDA-MB-231 (human breast adenocarcinoma) cell lines were purchased from American Type Culture Collection (ATCC; Manassas, VA, USA). The HMEC (human mammary epithelial cell) line was purchased from Lonza (Walkersville, MD, USA). Wild-type and poly(ADP-ribose) glycohydrolase (PARG)-null embryonic trophoblast stem (TS) cells were derived from E3.5 mouse blastocysts as previously described (18). They were maintained in growth medium containing fibroblast growth factor-4, heparin sodium, murine embryonic feeder-conditioned medium, 15% FBS, and 0.5 mM benzamide (an inhibitor of poly(ADP-ribose) polymerase). Dulbecco’s modified Eagle’s medium (DMEM) was purchased from Hyclone (Logan, UT, USA). Fetal bovine serum (FBS) was purchased from Atlas Biologicals (Fort Collins, CO, USA). Mammary epithelial growth medium (MEGM), which consists of mammary epithelial basal medium (MEBM) plus growth supplements (see ‘Cell culture’ below), was purchased from Lonza. Trypsin-EDTA (0.25%), penicillin-streptomycin solution, and glutamine were purchased from Invitrogen (Carlsbad, CA, USA).

### Other reagents

OptiMEM reduced serum medium and Lipofectamine 2000 reagent were purchased from Gibco Life Technologies (Grand Island, NY, USA). Protease inhibitor cocktail tablets (Complete Mini, EDTA-free) were purchased from Roche (Mannheim, Germany). Primary antibodies utilized were polyclonal rabbit anti-human TRPM2 antibody (Cat. #A300-414A, Bethyl Laboratories, Montgomery, TX, USA), polyclonal rabbit anti-human β-actin (Cat. #600-401-886, Rockland Immunochemicals, Limerick, PA, USA), polyclonal rabbit anti-human manganese superoxide dismutase (MnSOD) (Cat. #06-984, Millipore, Billerica, MA, USA), and monoclonal mouse anti-human Lamin B2 clone LN43 (Cat. #MA1-06104, Thermo Fisher Pierce, Pittsburgh, PA, USA). The secondary antibodies, horseradish peroxidase (HRP)-conjugated goat anti-rabbit and HRP-conjugated rabbit anti-mouse were purchased from Sigma (St. Louis, MO, USA). 2-Aminoethoxydiphenyl borate (2-APB), maintained as a 75 mM stock solution in dimethyl sulfoxide (DMSO), and 30% hydrogen peroxide solution were purchased from Sigma. The Fluo-4 NW Calcium Assay kit was purchased from Life Technologies. Comet Assay kit, which includes alkaline lysis solution, LMAgarose, 2-well CometSlides, SYBR Green, and EDTA, was purchased from Trevigen (Gaithersburg, MD, USA). CytoScan WST-1 cell proliferation assay was purchased from VWR International (Radnor, PA, USA).

### Cell culture

HEK 293, MDA-MB-231, and MCF-7 cells were grown and maintained in DMEM supplemented with 10% FBS, 100 U/ml penicillin/streptomycin, and 2 mM L-glutamine. Noncancerous human mammary cells (MCF-10A and HMEC) were cultured in MEGM specialty medium. MEGM consists of mammary epithelial cell basal medium (MEBM) plus the following growth supplements: 5 μg/ml bovine pituitary extract, 0.01 μg/ml human epidermal growth factor, 0.5 μg/ml hydrocortisone, GA-1000 (60 μg/ml gentamicin and 0.03 μg/mL amphotericin B), and 5 μg/ml insulin. All cultures were incubated at 37°C in 5% CO_2_ until treatments and analyses. Every two days in culture, cells were washed once with phosphate-buffered saline, pH 7.2 (PBS) and cultured in fresh growth medium.

### RNA interference

The silencing of TRPM2 by RNAi was performed as previously reported using siRNA specific to TRPM2 (5′-AUAGAUCAGGAACUCCGUCUC-3′) (17). This RNA oligo was purchased as duplexed RNA from Integrated DNA Technologies (Coralville, IA, USA). Each siRNA duplex was resuspended in RNase-free water at a final concentration of 40 μM and stored at −20°C. Universal scrambled control siRNA oligos were purchased as duplex RNA from Sigma and were used for all negative controls (19).

For RNAi transfections, cells were plated in 0.5 ml of medium per well without antibiotics in 24-well plates one day before transfection. At the time of transfection, cells were ~50% confluent. For each transfection, two mixtures were prepared: i) duplex siRNA added to 50 μl OptiMEM medium, and ii) 1 μl Lipofectamine 2000 added to 50 μl OptiMEM. After 5 min, the two solutions were gently mixed and then incubated at room temperature for 20 min. Final concentrations of siRNA were 100 nM for all cell lines. The mixtures were added drop wise to each well and cells were cultured for an additional 48–72 h.

### Whole cell lysate extraction

Cells were grown on 6-well tissue culture plates, harvested by trypsinization, washed once with 0.5 ml ice-cold PBS, and resuspended in 0.5 ml lysis buffer containing 25 mM Tris-HCl (pH 7.5), 150 mM NaCl, 1 mM EDTA, 1 mM EGTA, 1% NP-40, and protease inhibitors (Complete Mini, EDTA-free tablets). Suspensions were incubated for 30 min on ice and vortexed every 10 min. Cleared cell lysates were obtained after centrifugation at 16,000 × g for 10 min. Sodium dodecyl sulfate polyacrylamide gel electrophoresis (SDS-PAGE) sample buffer (50 mM Tris-HCl pH 6.8, 1% SDS, 2.5% glycerol, 0.005% bromophenol blue and 100 mM dithiothreitol) was added to the supernatants. Samples were then heated for 2 min at 95°C in a digital dry bath incubator (Labnet International, Edison, NJ, USA).

### Subcellular fractionations

Cells were grown in 60-mm tissue culture dishes (approximately 2×10^6^ cells/dish) and harvested by cell scraping in ice-cold PBS. After mild centrifugation (200 × g for 5 min), pellets were then fractionated using the NE-PER Nuclear and Cytoplasmic Extraction kit (Thermo Fisher Pierce, Rockfield, IL, USA) according to the manufacturer’s protocol. Briefly, harvested cells were washed with 1 ml ice-cold PBS and transferred to a 1.5 ml micro centrifuge tube. Cell pellets were obtained by centrifugation at 500 × g for 3 min at 4°C. The supernatant was removed and the pellet was resuspended in 0.2 ml ice-cold CER I solution containing protease inhibitors. The suspension was vortexed for 15 sec and placed on ice for 10 min. After addition of 11 μl of CER II solution, the suspension was vortexed for 5 sec, placed on ice for 1 min, and vortexed again. The extract was centrifuged at 16,000 × g for 5 min at 4°C and the supernatant, which represents the cytoplasmic fraction, was removed. The pellet, which contains the nuclei, was washed once with PBS as before and then resuspended in 0.1 ml of ice-cold NER solution. The nuclei were vortexed and placed on ice for 10 min. This was repeated 3 times for a total of 40 min. The extract was centrifuged as before for 10 min and the supernatant, which represents the nuclear fraction, was removed. Nuclear and cytoplasmic fractions were prepared in SDS-PAGE sample buffer and heated in a standard manner, as previously described.

### Immunoblotting

The protein concentration for all samples was obtained using the Pierce BCA Protein Assay kit (Thermo Scientific). Approximately 20 μg of each lysate or subcellular fraction sample was subjected to 7.5% SDS-PAGE. The proteins were transferred to 0.45 μm nitrocellulose by semi-dry transfer at 25 V for 1 h using a Trans-blot SD apparatus (Bio-Rad Laboratories, Hercules, CA, USA). Membranes were blocked with PBS containing 0.05% Tween-20 (PBST) and 5% milk at room temperature for 1 h and incubated with primary antibodies (1:1,000 anti-TRPM2, 1:3,000 anti-MnSOD, or 1:1,000 anti-Lamin B2) in PBST+5% milk overnight (shaking) at 4°C. Membranes were then washed with PBST three times and incubated with horseradish peroxidase (HRP)-conjugated goat anti-rabbit or HRP-conjugated rabbit anti-mouse antibody (1:10,000) for 1 h. The membranes were washed as described above, and chemiluminescence was initiated using the SuperSignal West Pico Chemiluminescent Substrate (Thermo Fisher Pierce). Immunoblots were then developed on a ChemiDoc XRS gel imaging system (Bio-Rad Laboratories). For quantification of protein levels for each blot, immunoblots were examined by densitometry with the ChemiDoc imager using Quantity One software. Relative densitometry ratios were then calculated using β-actin as loading controls. The resulting ratio of TRPM2/β-actin provided values that were then used to quantify relative protein levels.

### Proliferation assays

Cell proliferation assays using CytoScan WST-1 (Roche) were performed according to the manufacturer’s specifications. This assay measures cellular dehydrogenase activity, which is directly correlated to cell number. The reduction of a tetrazolium salt by cellular dehydrogenases is detected by spectrophotometer analysis at 425 nm. Briefly, cells were seeded at 5,000 cells per well in triplicate in a 96-well plate in growth medium appropriate to each cell type. Cells were incubated overnight and then treated with 100 μM 2-APB, scrambled siRNA or 100 nM TRPM2 siRNA the following day. At 0, 24, 48, 72 and 96 h time points, the media was removed, WST-1 reagent was added (10 μl WST-1 premixed with 100 μl growth medium per well), and cells were incubated for 1 h at 37°C. Analysis was performed using a BioTek Synergy HT microplate reader using Gen5 software (Winooski, VT, USA).

### Intracellular calcium measurement

A Fluo-4 NW Calcium Assay kit (Invitrogen) was used to measure intracellular calcium influx on a BioTek Synergy HT microplate reader following the manufacturer’s protocols. Briefly, approximately 5×10^4^ cells per well were grown in a 96-well plate overnight. The next day, plates were washed twice in Ca^2+^-free HBSS supplemented with HEPES buffer (pH 7.2), and then the growth medium was replaced with 100 μl/well of the Fluo-4 dye solution containing probenecid (to prevent extrusion of the dye out of the cells). The plate was incubated at 37°C for 30 min and then at room temperature for an additional 30 min in the dark. The loaded cells were then placed in the measurement position in a BioTek Synergy fluorescence spectrophotometer. Changes in fluorescence from the Fluo-4-NW dye quantify changes in intracellular Ca^2+^ concentrations (excitation/emission 485/535 nm) after treatment with 5 mM hydrogen peroxide (H_2_O_2_). Ca^2+^ influx was measured up to 40 min.

### Comet assays

Comet assays were performed using the CometAssay ES system from Trevigen. The manufacturer’s instructions for alkaline electrophoresis were followed. In brief, cells were seeded in 24-well tissue culture plates, incubated overnight in 0.5 ml growth medium, then treated the following day with 100 μM 2-APB or transfected with 100 nM TRPM2 siRNA. After collection by trypsinization 24 h later, a cell concentration of 1×10^5^ cells/ml was combined with molten low melting point agarose at a ratio of 1:10 (v/v). Agarose/cell mix (50 μl) was immediately pipetted onto a CometSlide and the slides were placed at 4°C in the dark for 30 min to allow solidification of the gel. The slide was then placed in lysis solution at 4°C overnight. The following day, the lysis buffer was removed and cells were immersed in freshly prepared alkaline unwinding solution for 20 min at room temperature. Cold alkaline electrophoresis solution was added to the CometAssay ES electrophoresis unit and slides were placed into the electrophoresis chamber. Horizontal electrophoresis was performed at 18 V for 30 min. When electrophoresis was complete, the slides were immersed twice in water and once in 70% ethanol for 5 min each, and then dried at 37°C for 15 min to bring all the cells into a single plane. The slides were then stored at room temperature with a desiccant until ready for analysis.

To stain the CometSlides, 100 μl of SYBR Green solution was added to each well and left for 30 min at room temperature, and then allowed to dry completely at 37°C. The slides were imaged using a Zeiss AxioObserver Z1 inverted fluorescence microscope (Thornwood, NY) with Hamamatsu Orca-ER digital camera and Axiovision software. Images were then analyzed using CometScore software (Tritek Corp., Sumerduck, VA, USA). A minimum of 200 cells for each treatment group were scored for quantification of ‘Percent DNA in Tail’, a standard comet assay value that represents DNA damage (20,21). This value was computed as the total comet tail intensity divided by the total comet intensity, multiplied by 100.

### Statistical analyses

All error bars for proliferation, protein levels, intracellular calcium influx, and comet assay quantifications represented the standard error of the mean (SEM). Statistical analyses were accomplished by one-way analysis of variance (ANOVA) followed by Tukey’s test and unpaired Student’s t-test. Statistical significance was defined as p<0.05.

## Results

### TRPM2 levels in noncancerous and cancerous breast cells

**Figure 1:**
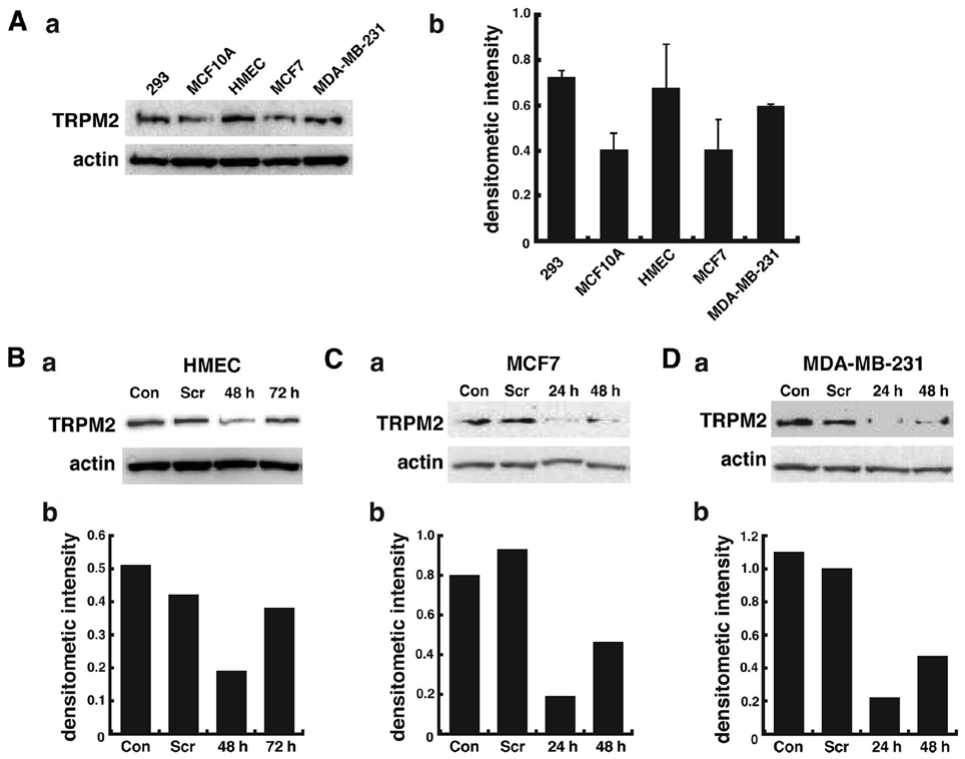
TRPM2 levels in breast cell lines and knockdown of TRPM2 levels by RNAi silencing. (A) Immunoblot (a) and densitometric quantification (b) of TRPM2 protein levels in two noncancerous breast cell lines (HMEC, MCF-10A) and two cancerous breast cell lines (MCF-7, MDA-MB-231). Immunoblot detection of TRPM2 protein levels in human embryonic kidney cells (HEK 293) provided the positive control. (B) RNAi silencing of TRPM2 in noncancerous HMEC cells (a), and quantification of protein levels by densitometry (b). (C and D) RNAi silencing of TRPM2 in MCF-7 and MDA-MB-231 breast adenocarcinoma cells (a) and quantification of protein levels by densitometry (b). All densitometric values represent TRPM2:actin ratios. Con, untreated negative control cells; Scr, treatment with scrambled negative control siRNA oligos.

Although TRPM2 channels are nearly ubiquitously expressed, we first analyzed the presence of TRPM2 channels in two lines of noncancerous human mammary epithelial cells and two lines of human breast adenocarcinoma cells by western blotting. The positive control for TRPM2 levels was provided by human embryonic kidney cells (HEK 293 cell line), as previous studies report the presence of TRPM2 mRNA (22) and endogenous protein expression (23) in these cells. Immunoblot analysis demonstrated significant levels of TRPM2 in HEK 293 cells, as expected (Fig. 1A). The results also show that TRPM2 levels in the two noncancerous mammary epithelial cells were variable, with greater levels in the HMEC cell line as compared to the MCF-10A cell line (Fig. 1A-a). In two lines of metastatic breast adenocarcinoma cells, greater levels of TRPM2 were observed in the MDA-MB-231 cell line as compared to the MCF-7 cell line. However, the increased levels of TRPM2 in HMEC cells (versus MCF-10A cells) and MDA-MB-231 cells (versus MCF-7 cells) as quantified by densitometry were not statistically significant (Fig. 1A-b). However, these results provide qualitative evidence that TRPM2 is present in each breast cell line. In summary, these results demonstrate that TRPM2 is present in two lines of noncancerous and two lines of cancerous breast cells.

### Effects of TRPM2 pharmacologic inhibition and TRPM2 RNAi silencing in breast adenocarcinoma cells

We utilized a TRPM2-specific siRNA sequence to knock down TRPM2 expression in breast cells. The siRNA sequence we utilized was previously shown to effectively knock down TRPM2 expression in both noncancerous prostate cells and prostate cancer cells (17). Using this siRNA sequence, we successfully decreased TRPM2 protein levels in noncancerous HMEC cells, where levels were decreased >60% as compared to control levels after 48 h (Fig. 1B). In breast adenocarcinoma cells after 48 h of RNAi silencing, TRPM2 levels were decreased more than 75% in the MCF-7 cell line (Fig. 1C) and ~80% in the MDA-MB-231 cell line (Fig. 1D) as compared to control levels. The results demonstrate the effective RNAi silencing of TRPM2 in noncancerous and cancerous human breast cells.

**Figure 2:**
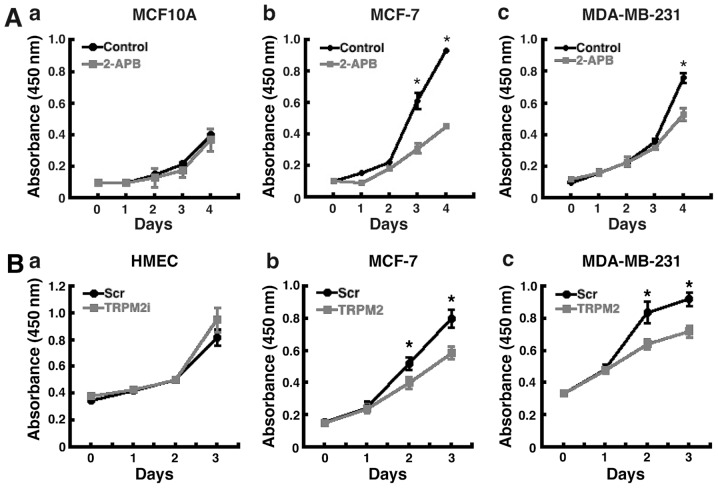
TRPM2 inhibition or RNAi knockdown of TRPM2 causes decreased breast adenocarcinoma cell proliferation. (A) Treatment of breast cell lines with the TRPM2 inhibitor, 2-aminoethoxydiphenyl borate (2-APB) (23) for 4 days and analysis of cell proliferation by CytoScan WST-1 assay. (B) RNAi silencing of TRPM2 and subsequent assay of cell proliferation up to 3 days. Gray plots represent 2-APB or TRPM2 RNAi-treated cells; black plots represent untreated or treatment with scrambled negative control oligos (Scr). cells. ^*^p<0.05 one-way ANOVA; error bars represent the standard error of the mean (SEM).

We next determined the effect of TRPM2 pharmacologic inhibition and RNAi silencing on breast adenocarcinoma cell proliferation. Treatment of the cells with 2-aminoethoxydiphenyl borate (2-APB), a pharmacologic inhibitor of TRPM2 (24), led to decreased proliferation in both lines of human breast adenocarcinoma cells (Fig. 2A-b and A-c). Treatment with 2-APB did not significantly effect the proliferation of noncancerous MCF-10A human breast epithelial cells (Fig. 2A-a). The effect was greater in MCF-7 cells, where decreased proliferation was evident by post-treatment day 3 (Fig. 2A-b). After 4 days of 2-APB treatment, proliferation was decreased nearly 60% in MCF-7 cells. Further, an effect was also observed in MDA-MB-231 breast adenocarcinoma cells, where proliferation was decreased ~40% after four days of 2-APB treatment (Fig. 2A-c). These results show that treatment with the TRPM2 inhibitor, 2-APB, leads to decreased proliferation in human breast adenocarcinoma cells, but not in noncancerous human mammary epithelial cells.

To verify that these effects were indeed due to the inhibition of TRPM2 function, the effect of TRPM2 RNAi silencing on cell proliferation was then analyzed. In both MCF-7 and MDA-MB-231 breast adenocarcinoma cells, decreased proliferation was observed 2 days after RNAi treatment (Fig. 2B-b and B-c). After 3 days, RNAi silencing of TRPM2 led to a 30–40% reduction in proliferation in these cells. No effect on proliferation was observed after TRPM2 RNAi silencing in noncancerous HMEC cells (Fig. 2B-a). These results thus demonstrate that the specific knockdown of TRPM2 led to decreased proliferation in human breast adenocarcinoma cells, but not in noncancerous human mammary epithelial cells. Taken together, the results of the pharmacologic inhibition of TRPM2 and RNAi silencing of TRPM2 indicate that TRPM2 has a role in facilitating the proliferation of human breast adenocarcinoma cells.

### Effect of TRPM2 inhibition on calcium influx in breast adenocarcinoma cells

**Figure 3:**
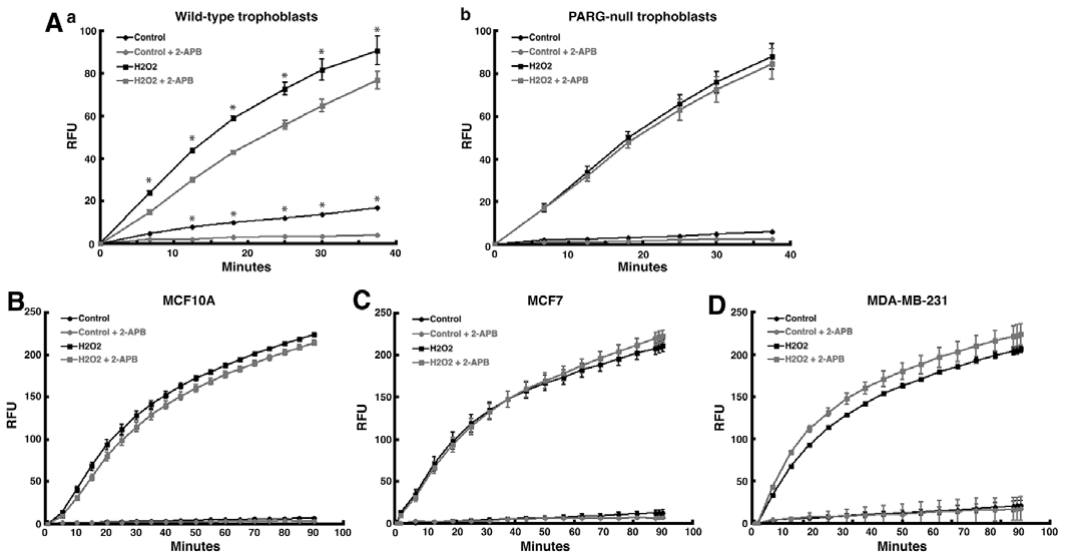
TRPM2 inhibition produces minimal changes in calcium influx in breast cancer cells after hydrogen peroxide treatment. (A) Pretreatment of wild-type trophoblast stem (TS) cells (a) and PARG-null TS cells (b) with 2-APB, and assay for calcium influx by Fluo-4 NW calcium assay. Treatment with 5 mM hydrogen peroxide (H_2_O_2_) was utilized to initiate oxidative stress and subsequent calcium influx, as previously performed (10). Assay for calcium influx in noncancerous breast (MCF-10A) (B), MCF-7 breast adenocarcinoma cells (C), and MDA-MB-231 breast adenocarcinoma cells (D) after H_2_O_2_ treatment. Changes in fluorescence from the Fluo-4-NW dye quantify changes in intracellular Ca^2+^ concentrations. ^*^p<0.05 one-way ANOVA; error bars represent the SEM.

TRPM2 is recognized as a plasma membrane ionophore, where it gates cations, including calcium, into the cell. Since TRPM2-mediated calcium gating is not well studied in breast cells, we again utilized 2-APB, as it was previously used to block the influx of cations by the TRPM2 channel in several cell lines (24). Thus, via the use of 2-APB, we analyzed the ability of TRPM2 channels to promote calcium influx in breast adenocarcinoma cells after oxidative stress. To analyze TRPM2 function, we utilized the Fluo-4 NW calcium assay to quantify calcium influx into breast adenocarcinoma cells after stimulation by hydrogen peroxide, as this assay was previously utilized to measure calcium influx due to TRPM2 channels (10). To validate the assay, we used the assay to measure TRPM2-mediated calcium influx in wild-type and poly(ADP-ribose) glycohydrolase (PARG)-null trophoblast stem (TS) cells. We used these particular cell lines because the primary molecule that activates TRPM2 channels is ADP-ribose. ADP-ribose, a product produced by the hydrolysis of poly(ADP-ribose) (PAR) polymers by PARG (25), binds TRPM2, opens the channel, and causes the gating of cations into the cell (6,26). Because of this, TRPM2-mediated calcium influx should be minimal in PARG-null TS cells, which contain no PARG enzymatic activity. In wild-type TS cells, treatment with hydrogen peroxide led to significant levels of calcium influx (Fig. 3A-a). Pretreatment of these cells with 2-APB caused significant decrease in calcium influx, which demonstrated that 2-APB blocked the influx of calcium by TRPM2 channels in wild-type TS cells. However, in PARG-null TS cells, pretreatment with 2-APB produced no significant effect on calcium influx following H_2_O_2_ treatment (Fig. 3A-b). This demonstrated that without PARG, ADP-ribose was not generated, the TRPM2 channel was not activated, and calcium was thereby not gated into the cell in PARG-null TS cells. Thus, the failure of 2-APB to decrease calcium influx in PARG-null TS cells validated the Fluo-4 NW calcium assay.

Of note, pretreatment with 2-APB failed to decrease the level of intracellular calcium stimulated by hydrogen peroxide in either MCF-7 or MDA-MB-231 human breast adenocarcinoma cells (Fig. 3C and D). Further, intracellular calcium influx was greater in MDA-MB-231 cells pretreated with 2-APB (Fig. 3D). In noncancerous MCF-10A breast epithelial cells, pretreatment with 2-APB decreased calcium influx stimulated by H_2_O_2_ at earlier time points (<15 min), while no statistical difference was observed at later time points (Fig. 3B). These results show that the pharmacologic inhibition of TRPM2 channels in human breast adenocarcinoma cells does not decrease calcium influx. This suggests that TRPM2 may not primarily function as a calcium channel in human breast adenocarcinoma cells, which further suggests that the role for TRPM2 in breast cancer cells may be distinct from its role in noncancerous cells.

### Nuclear localization of TRPM2 in human breast adenocarcinoma cells

**Figure 4:**
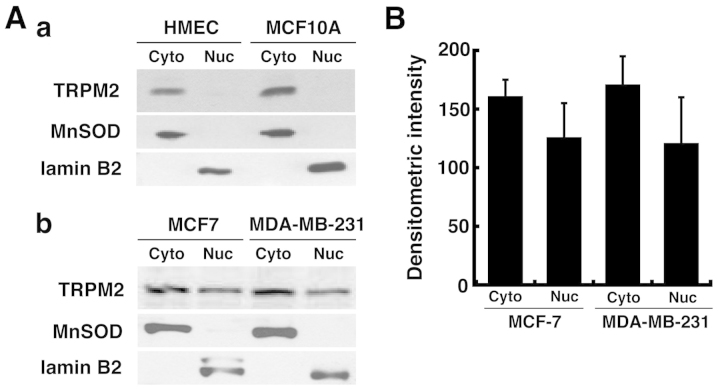
Nuclear localization of TRPM2 in breast cancer cells. (A) Noncancerous breast cell lines, HMEC and MCF-10A (a), and cancerous breast cell lines, MCF-7 and MDA-MB-231 (b), were fractionated into subcellular fractions (NE-PER Extraction kit, Pierce) and cellular localization of TRPM2 was then analyzed by immunoblot. Immunoblot detection of mitochondrial manganese superoxide dismutase (MnSOD) and nuclear lamin B2 was performed to verify successful fractionations. (B) Quantification of protein levels of TRPM2 in the cytoplasmic (Cyto) and nuclear (Nuc) fractions of MCF-7 and MDA-MB-231 cells by densitometry. Quantification is based on three immunoblots. Error bars represent SEM.

Because of the possibility that TRPM2 may have a novel role in human breast adenocarcinoma cells, our next objective was to determine its intracellular localization. In noncancerous cells, TRPM2 is normally localized to the plasma membrane as a non-specific cation channel (7). However, it has also been found to be localized to the lysosomal membrane (12). Thus, present studies show that TRPM2 has an extra-nuclear localization in normal cells. In agreement with these studies, our TRPM2 localization analyses demonstrated that TRPM2 has an extra-nuclear localization in noncancerous HMEC and MCF-10A breast cells (Fig. 4A-a), as TRPM2 protein was observed in the cytoplasmic fractions of these cells after subcellular fractionations. However, in MCF-7 and MDA-MB-231 human breast adenocarcinoma cells, TRPM2 was present in the nuclear fractions of these cells (Fig. 4A-b). This localization was not exclusive, as TRPM2 was also observed in the cytoplasmic fractions in these cells. Quantification of protein levels by densitometry indicated that ~40–45% of the TRPM2 protein present in human breast adenocarcinoma cells was localized to the nucleus (Fig. 4B). As these results are consistent with the nuclear TRPM2 localization in prostate cancer cells shown previously (17), our data thus demonstrate that TRPM2 is present in the nuclei of MCF-7 and MDA-MB-231 human breast adenocarcinoma cells. This suggests that TRPM2 may have a novel role in the nuclei of these cells.

### Inhibition or RNAi silencing of TRPM2 causes increased DNA damage in breast adenocarcinoma cells

**Figure 5:**
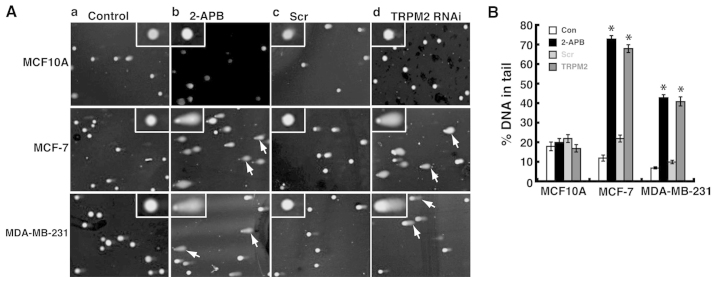
TRPM2 inhibition and RNAi knockdown of TRPM2 causes increased DNA damage in breast adenocarcinoma cells. (A) Breast cells were pretreated with 2-APB or RNAi silencing of TRPM2, then analyzed for DNA damage by comet assay. White arrows point to comet tails, which signify DNA damage. Insets located in the upper right or upper left of each panel contain a representative cell from each treatment group that is magnified to allow a closer visualization of comets. (B) Quantification of comet assay using CometScore software. Because DNA in the tail of the comets represents DNA damage, a common method to quantify comet assay results is to calculate the ‘percent DNA in tail’. This value provides a direct correlation to the amount of DNA damage per treatment group. A minimum of 200 cells per treatment group were analyzed for each quantification. ^*^p<0.05 one-way ANOVA; error bars are SEM.

To investigate a possible nuclear role of TRPM2 in human breast adenocarcinoma cells, we determined the effect of TRPM2 pharmacologic inhibition and TRPM2 RNAi silencing on the levels of DNA damage in MCF-7 and MDA-MB-231 cells. This was performed by utilizing the single cell gel electrophoresis (comet) assay. The comet assay is based on the alkaline lysis of labile DNA at sites of damage (27). The unwound, relaxed DNA is able to migrate out of the cell during electrophoresis and can be visualized using SYBR green nucleic acid stain. Cells that have accumulated DNA damage appear as fluorescent comets with tails that represent DNA fragmentation. In noncancerous human MCF-10A breast epithelial cells, pretreatment with 2-APB did not cause the formation of significant levels of comets (Fig. 5A-b), which indicates minimal levels of DNA damage. However, in MCF-7 human breast adenocarcinoma cells, significant levels of comets were observed after 2-APB treatment (Fig. 5A-b) as compared to untreated cells (Fig. 5A-a). Similar results were observed in MDA-MB-231 human breast adenocarcinoma cells, where 2-APB treatment led to significant amounts of comets as compared to untreated cells (Fig. 5A-a and A-b). These results demonstrated that treatment with the TRPM2 inhibitor, 2-APB, led to increased levels of DNA damage in human breast adenocarcinoma cells.

To verify that these effects were due to the inhibition of TRPM2, we performed TRPM2 RNAi silencing in these cells. RNAi silencing of TRPM2 in noncancerous MCF-10A cells did not lead to significant numbers of cells with comets (Fig. 5A-d). However, in both MCF-7 and MDA-MB-231 breast adenocarcinoma cells, RNAi silencing of TRPM2 led to increased amounts of cells with comets as compared to cells transfected with negative control scrambled siRNA oligos (Fig. 5A-c and A-d). These results verify that the increased level of DNA damage in human breast adenocarcinoma cells is mediated via the inhibition or knockdown of TRPM2. Since DNA in the tail of the comets represents DNA damage, a common quantification of comet assay results is the ‘percent DNA in tail’ (20). Thus, using CometScore software and analyzing a minimum of 200 cells per treatment group, we calculated this value for the human breast cell lines treated with 2-APB or RNAi silencing of TRPM2. The resulting values demonstrated that increased levels of DNA damage were quantified in MCF-7 and MDA-MB-231 cells as compared to noncancerous control cells (Fig. 5B). Further, there appears to be even greater levels of DNA damage in MCF-7 cells as compared to MDA-MB-231 cells. This is seen through the ‘percent DNA in tail’ values for 2-APB treated or TRPM2 RNAi-silenced MCF-7 cells, which were ~70% each, as compared to ~40% in MDA-MB-231 cells (Fig. 5B). No significant increases in percent DNA in tail values for 2-APB treated or TRPM2 RNAi-silenced MCF-10A cells were observed, which demonstrates minimal effects of TRPM2 inhibition or knockdown on DNA damage levels in noncancerous human mammary epithelial cells. Taken together, the results show that the pharmacologic inhibition or RNAi silencing of TRPM2 led to increased levels DNA damage in human breast adenocarcinoma cells. This indicates that TRPM2 has a protective role in these lines of human breast adenocarcinoma cells, where it somehow minimizes damage to genomic DNA.

## Discussion

This study provides insight into the role of TRPM2 in breast cancer cells that potentially provides a foundation for investigating TRPM2 inhibition to selectively target the DNA of breast adenocarcinoma cells in the future. The discovery of a unique role for TRPM2 in breast cancer cells, along with the ability of TRPM2 inhibition or RNAi silencing to increase DNA damage and decrease cell proliferation specifically in breast cancer cells, suggests that the pharmacologic inhibition of TRPM2 may specifically target breast tumors. Further, current studies that show protective effects in noncancerous cells due to TRPM2 inhibition (7–9), along with our studies here that show the absence of harmful effects in noncancerous breast cells after TRPM2 inhibition or RNAi silencing, suggest that pharmacologic agents that inhibit TRPM2 are expected to produce deleterious effects only in breast cancer cells. Thus, our study provides the preliminary results necessary to further study the ability of TRPM2 pharmacologic inhibition to prevent the survival, proliferation, and/or metastasis of breast adenocarcinoma cells.

Our results are in agreement with a previous study that utilized prostate cancer cell lines (17). This previous study demonstrated a nuclear localization of TRPM2 in prostate cancer cells. RNAi knockdown of TRPM2 decreased the proliferation of these cells. Further, a nuclear localization and decreased proliferation were not observed in noncancerous prostate cells. While the authors of this study presented these effects in prostate cancer cells, we provide here the first study that demonstrates such effects in breast adenocarcinoma cells. Further, we expand upon these findings by identifying a novel protective effect of TRPM2 in breast adenocarcinoma cells. Loss of TRPM2 function causes significant increases in DNA damage. This function is distinct from its function in non cancerous cells. Because this role involves genomic DNA, it thus appears that the nuclear localization of TRPM2 facilitates its ability to somehow provide protective effects toward genomic DNA. Future studies will be required to determine exactly how TRPM2 accomplishes this function. Possibilities include the facilitation of DNA repair by nuclear TRPM2 or the stimulation of Ca^2+^-mediated functions in the nucleus by promoting nuclear calcium influx.

A recent study demonstrated a protective role for TRPM2 in cardiac myocytes in response to reperfusion injury (28). This is in contrast to the role of TRPM2 in noncancerous cells, where the activation of cation gating by TRPM2 exacerbates injury in response to oxidative stress. Further, it is potentially consistent with our studies that show a protective role for TRPM2 in breast cancer cells. However, this study demonstrated the ability of TRPM2 to facilitate mitochondrial bioenergetics and electron transport in cardiac cells. Also, no nuclear localization of TRPM2 was shown in this study. It is possible that the effects of TRPM2 inhibition on genomic DNA damage in breast cancer cells are partially due to effects on the mitochondria. However, the lack of deleterious effects in noncancerous breast cells provides evidence against the ability of TRPM2 inhibition to disrupt mitochondrial function and promote reactive oxygen species generation in breast cells. It is thus possible that TRPM2 may have different roles in cardiac cells and breast cancer cells.

We show here minimal effects of TRPM2 inhibition on calcium influx in breast cancer cells. This suggests that TRPM2 may not exclusively function as a cation channel in breast cancer cells. However, it is possible that TRPM2 may have a lesser role as an ionophore or ion channel, as TRPM2 was shown to also be localized to the cytoplasmic fraction in breast cancer cells. Further, the abundance of TRPM2 protein levels in breast cancer cells may be significantly decreased as compare to their levels in neurons and developmental cells, where TRPM2-mediated calcium influx is robust. Moreover, it is possible that TRPM2 may control the influx of calcium in the nucleus. Further studies will be necessary to determine if TRPM2 mediates significant levels of calcium influx into the cytoplasm or nuclei of breast adenocarcinoma cells.

Future studies may also include the investigation of the ADP-ribose pyrophosphatase enzymatic activity of TRPM2 channels in breast cancer cells. Since this ability is a unique feature of TRPM2 channels, it may prove to be significant in maintaining the survival, proliferation, or limiting DNA damage in breast adenocarcinoma cells. For example, the hydrolysis of the nucleotide, ADP-ribose, may facilitate prolonged survival in breast cancer cells via a nucleotide signaling pathway. These studies, along with the aforementioned calcium influx studies, are important, because they will provide the necessary information to determine if preventing TRPM2 cation gating (i.e. inhibiting the ion channel) or antagonizing the enzymatic activity of TRPM2 is the key determinant for successfully targeting the DNA of breast adenocarcinoma cells. Thus, this present study, along with these future investigations, may provide the foundation necessary for the initial rational drug design of TRPM2 inhibitors to be used in the treatment of breast cancer.

In summary, we discovered a novel role for TRPM2 in breast adenocarcinoma cells. This role appears to be essential for these cells to survive and proliferate. Thus, this study fits with a new paradigm for cancer drug development that identifies a vulnerability that can lead to agents with a much higher therapeutic index because of greatly reduced general toxicity. Indeed, our data showing that reducing TRPM2 activity is not toxic in normal cells, but is toxic in breast cancer cells, supports the new paradigm leading to new cancer drugs. As there is growing evidence that specific types of cancer contain specific vulnerabilities, the presence of nuclear TRPM2 may therefore represent one such vulnerability.

## Abbreviations

2-APB: 2-aminoethoxydiphenyl borate
ADP-ribose: adenosine diphosphoribose
MEBM: mammary epithelial basal medium
MEGM: mammary epithelial growth medium
MnSOD: manganese superoxide dismutase
PAR: poly(ADP-ribose)
PARG: poly(ADP-ribose) glycohydrolase
PARP: poly(ADP-ribose) polymerase
TRP: transient receptor potential superfamily of cation channels
TRPM: transient receptor potential, melastatin subfamily
TS cell: trophoblast stem cell
